# COVID-19: An urgent opportunity to decrease financial barriers to medical school admissions

**DOI:** 10.36834/cmej.70219

**Published:** 2020-12-07

**Authors:** Justin Lam, Chantal Phillips, Ike Okafor

**Affiliations:** 1 Faculty of Medicine, University of Toronto, Ontario, Canada; 2 Hospital for Sick Children, Ontario, Canada

In recent years, medical schools have recognized that achieving a diverse student body representative of the communities they serve is key to reducing population health disparities.^1^ Medical schools have implemented programs to reduce barriers to admission for students from under-represented backgrounds, but students from low socioeconomic status (SES) backgrounds still face financial barriers. COVID-19 has exacerbated many existing societal inequities and has also resulted in adjustments across the country to make interview processes more accessible. It is important to ensure these changes reduce, rather than reinforce, inequitable barriers to admission for students from low SES backgrounds.

The cost of applying to medical school has historically contributed to the disproportionate underrepresentation of students from low SES backgrounds, with interviews posing a particularly significant financial challenge in the application process.^2^ In 2018, nearly 30% of applicants had interview-related expenses of $1,500 USD or more in the United States.^2^ In the Canadian context, the substantial barriers posed by MCAT fees have been demonstrated,^3^ but to our knowledge, the impact of interview costs or application costs as a whole has not been studied. With applicants from the lowest household-income quintile earning approximately $2,000 USD and $2,200 CAD per month in the States^2^ and Canada respectively,^4^ applicants from low SES backgrounds may be unfairly required to choose between interviews or living expenses. Despite this disparity, measures to reduce the cost of medical school admissions interviews have not been implemented.

Because of COVID-19, existing ideas for increasing accessibility to interviews have suddenly been implemented. For example, medical schools have widely adopted interviews via videoconference or had students submit their recorded responses to emailed interview questions. Interviews over videoconference can eliminate costs associated with travel and lodging and are more convenient to schedule. However, they may still disadvantage candidates from low SES backgrounds who might not have a private room or a reliable internet connection for their interviews. This interview format also reduces opportunities for interaction with potential future classmates and faculty. Although equivalence between in-person and videoconferencing interviews has been reported,^5^ it is important for institutions to explore local applicability given variations in interview formats. Therefore, when considering the long-term application of this practice, it is essential to collect the experiences of all stakeholders in the admissions process to monitor for and address any unintended consequences.

Medical schools have a responsibility to train physicians who reflect the communities they serve. Addressing financial barriers in the admissions process is imperative to achieving this goal. The COVID-19 pandemic has forced adaptations to the interview process that make this increasingly possible. As the pandemic continues to affect income, other interventions to promote financial equity in our admissions processes are still necessary, such as reducing or eliminating application fees for students with financial difficulty, and reconsidering the MCAT requirement. Given the economic impact of COVID-19, lowering both direct and indirect application expenses is an urgent and pressing matter, aligned with Canadian medical schools’ commitment to diversity and social accountability. It is critical that the effectiveness of these changes be evaluated to make admissions processes more equitable during, but especially after, this pandemic.
